# Identification of a Resistance Mechanism to IGF-IR Targeting in Human Triple Negative MDA-MB-231 Breast Cancer Cells

**DOI:** 10.3390/biom11040527

**Published:** 2021-04-01

**Authors:** Jennifer Tsui, Shu Qi, Stephanie Perrino, Matthew Leibovitch, Pnina Brodt

**Affiliations:** 1 Department of Medicine, McGill University, Montreal, QC H4A 3J1, Canada; jennifer.tsui3@mail.mcgill.ca; 2 Division of Experimental Medicine, McGill University, Montreal, QC H4A 3J1, Canada; 3 McGill University Health Center Research Institute, Montreal, QC H4A 3J1, Canada; qishu2014@hotmail.com (S.Q.); stephanie.perrino@affiliate.mcgill.ca (S.P.); matthewleibovitch@hotmail.com (M.L.); 4 Department of Surgery, McGill University, Montreal, QC H4A 3J1, Canada; 5 Department of Oncology, McGill University, Montreal, QC H4A 3J1, Canada

**Keywords:** triple negative breast cancer, IGF signaling, the IGF-Trap, drug resistance, FGFR1

## Abstract

Triple negative breast cancer (TNBC) is associated with unfavorable prognosis and high relapse rates following chemotherapy. There is an urgent need to develop effective targeted therapy for this BC subtype. The type I insulin-like growth factor receptor (IGF-IR) was identified as a potential target for BC management. We previously reported on the production of the IGF-Trap, a soluble IGF-1R fusion protein that reduces the bioavailability of circulating IGF-1 and IGF-2 to the cognate receptor, impeding signaling. In nude mice xenotransplanted with the human TNBC MDA-MB-231 cells, we found variable responses to this inhibitor. We used this model to investigate potential resistance mechanisms to IGF-targeted therapy. We show here that prolonged exposure of MDA-MB-231 cells to the IGF-Trap in vitro selected a resistant subpopulation that proliferated unhindered in the presence of the IGF-Trap. We identified in these cells increased fibroblast growth factor receptor 1 (FGFR1) activation levels that sensitized them to the FGFR1-specific tyrosine kinase inhibitor PD166866. Treatment with this inhibitor caused cell cycle arrest in both the parental and resistant cells, markedly increasing cell death in the latter. When combined with the IGF-Trap, an increase in cell cycle arrest was observed in the resistant cells. Moreover, FGFR1 silencing increased the sensitivity of these cells to IGF-Trap treatment in vivo. Our data identify increased FGFR1 signaling as a resistance mechanism to targeted inhibition of the IGF-IR and suggest that dual IGF-1R/FGFR1 blockade may be required to overcome TNBC cell resistance to IGF-axis inhibitors.

## 1. Introduction

Breast cancer is the second most common cancer overall, and the most common cancer type in women worldwide, affecting millions of women annually [1]. Fifteen to twenty percent of all breast cancer cases are diagnosed as triple negative breast cancer (TNBC). Clinically, TNBCs are defined as tumors that are estrogen receptor (ER) and progesterone receptor (PR)-negative and lack HER2 overexpression or amplification, as determined by immunohistochemistry and in situ hybridization, respectively [2,3,4,5]. TNBCs are more frequently diagnosed in younger (<40 years of age), African American and Hispanic women [6,7,8,9]. Six distinct transcriptional molecular subtypes that harbor different pathway enrichments have been identified in TNBC [10], reflecting the complexity of this disease subtype. Unlike the hormone receptor and HER2-positive BC, there is currently no approved targeted therapy for TNBC due to the intra- and inter-tumor heterogeneity in this subtype, and chemotherapy remains, therefore, the primary treatment option [3,5]. Due to its aggressive nature and lack of treatment options, TNBC patients, in general, have poorer overall survival, shorter disease-free survival and increased risk of relapse within the first 3 years following initial diagnosis compared to patients with other breast cancer subtypes [6,11]. There is, therefore, an unmet urgent need to develop and optimize targeted therapy for TNBC.

The insulin-like growth factor (IGF) axis plays a critical role in normal development and physiology by conveying survival and growth signals through the PI3K/Akt and Ras/Raf/ERK pathways [12,13,14,15]. Malignant progression is often associated with upregulated insulin-like growth factor receptor (IGF-1R) expression and/or signaling as documented in diverse cancer types, including TNBC [14,16,17]. IGF-1 could enhance the survival and proliferation of TNBC cells in vitro, and IGF-1R/insulin receptor (IR) tyrosine kinase inhibitors (TKI) caused the regression of TNBC xenografts in vivo [18]. Moreover, increased IGF-1R signaling was observed in surgical TNBC specimens compared to normal breast tissue [19]. A study by Law et al. reported that 41.9% of TNBC specimens analyzed expressed activated IGF-1R and IR [20]. Moreover, IGF-1 and IGF-1R overexpression were associated with increased incidence of metastases and decreased survival in TNBC patients [21]. Collectively, these findings strongly implicated IGF-1R signaling in TNBC pathogenesis and identified the IGF axis as a potential target in this disease.

Despite this strong evidence for IGF-1R involvement in TNBC progression, results from clinical trials evaluating IGF-1R targeting monoclonal antibodies (MAb) and small molecule IGF-1R/IR TKI have been discouraging. This lack of clinical efficacy has been attributed to several factors including compensatory growth stimulation via IGF-2/IR-A signaling [22,23] and the undesirable toxicities such as hyperinsulinemia and hypoglycemia associated with TKI, due to their deleterious effects on the IR axis [24].

We previously reported on the production, optimization and characterization of the IGF-Trap, a soluble fusion protein comprised of the extracellular domain of human IGF-1R fused to the Fc portion of human IgG_1_ that binds IGF-1 and IGF-2 with a 10^3^-fold higher affinity than insulin [25,26]. The IGF-Trap reduced the bioavailability of circulating and locally produced IGF-1/2, thereby reducing IGF-1R signaling [25] (Reviewed in [27]). IGF-neutralizing agents with low affinity for insulin may have superior therapeutic profiles compared to anti-IGF-1R MAbs and TKIs because they also reduce the bioavailability of IGF-2, thereby limiting IGF-2/IR-A signaling, while also bypassing any potential deleterious effects on the IR/insulin axis. Previously, we found that in mice treated with the IGF-Trap, xenografts of the human TNBC MDA-MB-231 cells initially regressed [26]. However, in some mice, tumor recurrence was observed over time. This suggested that MDA-MB-231 cells may be heterogeneous in respect to sensitivity to IGF-1R signaling blockade and/or that some cells acquired resistance to IGF-inhibition over time. Here, we utilized this model to identify resistance mechanism(s) that could provide alternative growth signals, thereby rendering TNBC insensitive to IGF-signaling blockade and found that autocrine fibroblast growth factor receptor 1 (FGFR1) signaling conferred resistance to IGF-1R blockade.

## 2. Material and Methods

***Cells***. The human breast cancer cell line MDA-MB-231 was a generous gift from Dr. Peter Siegel (The Goodman Cancer Center, McGill University, Montreal, QC, Canada). These cells and all genetically modified variants were cultured in Dulbecco’s Modified Eagle Medium (DMEM) supplemented with 10% fetal bovine serum (FBS) and 1% penicillin-streptomycin (10,000 U/mL) and puromycin (1 μg/mL), where required. All cells were routinely mycoplasma-tested throughout this study.

***Mice***. Female NOD SCID IL2gammaR (NSG) mice were used for the in vivo experiments. NOD.Cg-Prkdcscid Il2rgtm1Wjl/SzJ Stock No: 005557 breeders were initially purchased from the Jackson Laboratory (Bar Harbor, ME, USA) and bred in-house at the animal facility of the Research Institute of the McGill University Health Center. Eight–twelve-week-old mice were used for all the experiments. 

***Reagents***. The bioengineering, cloning, production, and characterization of the IGF-Trap were described in detail previously [25,26,27]. Third generation IGF-Trap 3.1 was used in this study [25]. The rabbit anti IGF-1R MAb was from Abcam (Cambridge, MA, USA). Rabbit monoclonal antibodies to pIGF-IR and pAKT were from Cell Signaling Technology (Danvers, MA, USA). The monoclonal mouse antibody to β-actin was from Sigma Aldrich (Oakville, ON, Canada). The rabbit polyclonal antibodies to pERK, ERK, Akt, FGFR1, pFGFR1, S6K, pS6K, STAT3 pSTAT3 and caspase 3 were all from Cell Signaling Technology, and PD166866, a small molecule FGFR1 TKI, was from Sigma Aldrich. The optimal dose for each lot of this inhibitor was determined in preliminary dose response assays prior to use in the indicated analyses.

***Generating an IGF-Trap-resistant MDA-MB-231 subpopulation***. MDA-MB-231 cells were cultured in 24-well plates in complete DMEM medium containing increasing concentrations (40–315 µg/mL) of IGF-Trap. These concentrations were determined empirically based on preliminary assays to determine the optimal inhibitory IGF-Trap concentration. The range was calculated based on an estimated combined IGF-1/IGF-2 concentration of 50–90 µg/mL in 10% FBS medium [28]. The IGF-Trap was replenished every 48 h. When surviving cells reached confluency during 3–4 passages in the presence of a specific IGF-Trap concentration, the concentration was increased 2 fold. This process was repeated until resistance to the highest dose of 315 μg/mL IGF-Trap was observed. A batch of cells that remained resistant to this dose over time was frozen and aliquots used in all the experiments described. Hereafter, this subline is referred to as MDA-MB-231-R.

***RT-PCR and qPCR***. Cells were cultured in complete media (DMEM + 10% FBS) for 48 h. Total cellular RNA was extracted using Trizol (Life Technologies, Burlington, ON, Canada) and PCR was performed using standard procedures and the primers listed in Table S1. Amplified products were analyzed using electrophoresis (2% agarose gel) and visualized using the ImageQuant LAS 4000 imager (GE Healthcare Life Sciences, Baie d’Urfe, QC, Canada). The qPCR was performed using a standard protocol as previously described [29,30]. Amplification of the cDNA was performed using FastStart Universal SYBR Green Master (Rox) (Roche, Laval, QC, Canada) and a PCR mixture containing 0.8 μM of each of the indicated primers (Table S1) and 2 μL of cDNA in a LightCycler (Bio-Rad Laboratories, Mississauga, ON, Canada). The data were analyzed using the iQ5 software (Bio-Rad Laboratories).

***Western blotting***. Western blotting was performed using a standard protocol [29,30,31]. Proteins were resolved on 6–10% SDS-polyacrylamide gels under reducing conditions, transferred onto Polyvinylidene difluoride (PDVF)/nitrocellulose membranes, and probed, first at 4 °C overnight with the indicated primary antibodies and then at RT (room temperature) for 2 h with horseradish peroxidase (HRP)-conjugated secondary antibodies (1:10,000). Bands were visualized using the Amersham ECL prime/select detection reagent (GE Healthcare Life Sciences, Marlborough, MA, USA). Primary antibody dilutions were as follows: antibodies to pIGF-1R, IGF-1R, pFGFR1 and FGFR1 at 1:500, antibodies to pERK, ERK pAKT, AKT, S6K, pS6K, STAT3 and pSTAT3 and caspase 3 at 1:1000, anti GAPDH at 1:5000 and anti β-actin at 1:2000.

***RTK arrays***. MDA-MB-231 and MDA-MB-231-R cells were seeded in 6-well plates and allowed to adhere overnight. They were then serum starved overnight, treated with 315 μg/mL IGF-Trap in complete medium for 72 h, lysed and 300 μg of total cell lysates analyzed using the Proteome Profiler™ Human Phospho-RTK antibody arrays kit, catalogue # ARY001B (R&D Systems, Burlington, ON, Canada).

***IGF-IR activation***. Cells were cultured in complete medium overnight, serum starved for 24 h, stimulated with 100 ng/mL IGF-1 for the indicated time intervals in the presence or absence of IGF-Trap (2:1 ratio; IGF-Trap:IGF-1) and lysed using RIPA buffer (50 mM Tris, 150 mM NaCl, 0.1% SDS, and 1% Triton) containing a protease inhibitor cocktail (Roche), sodium orthovanadate and sodium pyrophosphate, prior to Western blotting.

***Cell proliferation/viability assays***. Cell proliferation was analyzed using the MTT (3-(4,5-Dimethylthiazol-2-yl)-2,5-Diphenyltetrazolium Bromide) assay or using trypan blue dye exclusion. Cells seeded in 48 or 96 well plates (10^4^ or 3 × 10^3^ cells/well for trypan blue exclusion and MTT assay, respectively) were serum starved overnight and treated with the indicated concentrations of the IGF-Trap and/or PD166866 in the presence of serum for the indicated time intervals. The MTT and dye exclusion assays were performed using standard procedures [26].

***Cell Cycle analysis***. Cell cycle was analyzed by flow cytometry. Cells in 6-well plates (5 × 10^4^ cells/well) were serum-starved overnight before treatment for 48–72 h with the indicated concentrations of PD166866, IGF-Trap, both or the vehicle (DMSO in medium), in the presence of serum. Media and cells were collected, centrifuged at 1000 rpm for 5 min, washed twice with phosphate-buffered saline (PBS) and fixed for 20 min in chilled 70% ethanol. Cells were stained with propidium iodide (20 μg/mL), treated with RNase A (100 μg/mL) for 2 h at 4 °C in the dark and debris removed using a cell strainer (100 μm) before the analysis. Cell cycle distribution was determined by acquiring at least 10^4^ events using the FACSCanto (BD Biosciences, San Jose, CA, USA) and the data were analyzed using the ModRFit software (Verity Software House, Topsham, ME, USA).

***FGFR1 silencing***. The pLKO.1-puro lentiviral FGFR1 short hairpin RNA (shRNA) and non-mammalian shRNA clones were provided by the Genetic Perturbation Service of the Goodman Cancer Research Centre and Department of Biochemistry of McGill University. Three shRNA clones were evaluated, and the most effective (TRC clone ID: TRCN0000121182, https://portals.broadinstitute.org/gpp/public/clone/search, accessed on 17 September 2019) was used for transfection. In brief, plasmids containing the shRNA and packaging vectors were introduced into HEK293T cells using the X-tremeGENE transfection reagents (Sigma), according to the manufacturer’s instructions, and the virus produced in the culture supernatants was collected, concentrated and then added to MDA-MB-231 and MDA-MB-231-R cells along with 4 μg/mL polybrene. The transfected cells were incubated overnight, the medium refreshed, and the cells cultured for an additional 48 h, at which time the medium was replaced with fresh medium containing 5 μg/mL puromycin. Puromycin-resistant cells were obtained 2 weeks later and FGFR1 silencing verified by Western blotting before use of the cells in the experiments.

***Orthotopic tumor cell growth assay***. All mouse experiments were carried out in strict accordance with the recommendations of the Canadian Council on Animal Care (CCAC) and under the conditions and procedures approved by the Animal Care Committee of McGill University (AUP number: 5733). One million breast cancer cells in 0.05 mL Matrigel (BD Biosciences) diluted 1:1 with cold PBS were implanted in the mammary fatpads of female NSG mice. When tumors were established (50–100 mm^3^), the animals were randomized and injected i.v. with 5 mg/kg IGF-Trap, or vehicle, twice weekly until the mice were moribund. The tumors were measured twice weekly using calipers and tumor volumes were calculated using the formula: (4/3 × (3.14159) × (Length/2) × (Width/2)^2^).

***Statistical Analysis***. All results shown of in vitro analyses are based on at least three experiments or replicates. Data were analyzed using the one or two-tailed Student’s *t*-test or a one-way ANOVA.

## 3. Results

### 3.1. Ligand Induced IGF-1R Signaling in MDA-MB-231 Cells

**Figure 1:**
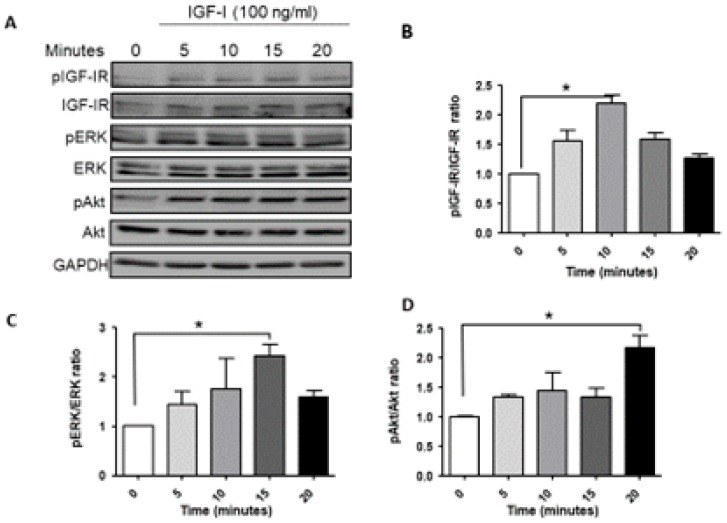
Ligand induced IGF-1R signaling in MDA-MB-231 cells. Cells were serum starved for 24 h and then stimulated with 100 ng/mL IGF-1 for the indicated time intervals. Shown in (**A**) is a representative Western blot of the IGF-IR and downstream signaling mediators following IGF-1 stimulation. Shown in the bar graphs (**B**–**D**) are the mean (and SE (standard error)) of 3 experiments expressed as pIGF-1R/IGF-1R, pERK/ERK, and pAkt/Akt ratios, respectively, normalized to the levels of unstimulated cells that were assigned a value of 1. * *p* < 0.05.

We began by analyzing the signaling activated downstream of ligand-induced IGF-1R activation in MDA-MB-231 cells. Serum starved cells were stimulated with 100 ng/mL IGF-1 and the cell lysates were analyzed by Western blotting. Ligand-induced IGF-1R activation was observed within 5 min, peaking at 10 min and still detectable at 20 min post stimulation (Figure 1A,B). A basal level of phosphorylated ERK was observed in these cells, consistent with the presence of *BRAF* and *KRAS* mutations [10]. IGF-1 increased ERK phosphorylation, as evident 5 min post stimulation, with activation levels peaking at 15 min (Figure 1A,C). Akt phosphorylation was also evident at 5 min, increasing progressively and maintained for 20 min post stimulation (Figure 1A,D). Together, these results confirmed that MDA-MB-231 cells were responsive to IGF-1, and both MEK/ERK and PI3K/Akt signaling were activated.

### 3.2. The IGF-Trap Blocks IGF-1 Signaling and Inhibits MDA-MB-231 Proliferation

**Figure 2:**
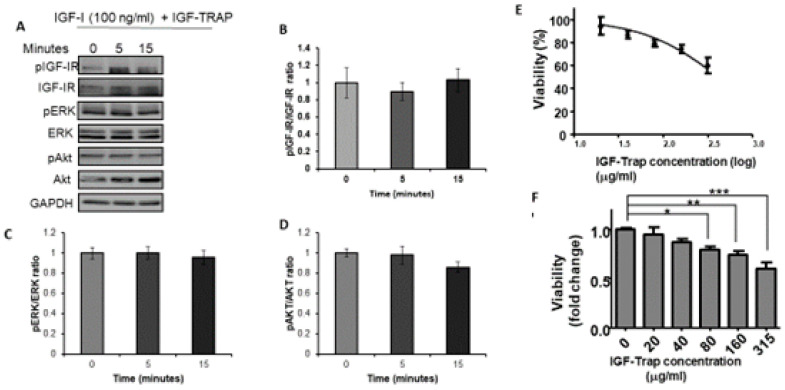
The IGF-Trap blocks IGF-1R signaling and inhibits the proliferation of MDA-MB-231 cells. Cells were serum starved for 24 h and then stimulated with 100 ng/mL IGF-1 in the presence of IGF-Trap added at a molar ratio of 2:1 (IGF-Trap:IGF-1) for the indicated time intervals. Shown in (**A**) is a representative Western blot of the IGF-1R and downstream signaling mediators following IGF-1 stimulation. Shown in the bar graphs (**B**–**D**) are the means (±SE) of 3 experiments expressed as pIGF-1R/IGF-1R, pERK/ERK, and pAkt/Akt ratios, respectively, normalized to the levels of unstimulated cells that were assigned a value of 1. The anti-proliferative effect of the IGF-Trap was measured by the MTT assay. Cells were treated with the indicated concentrations of the IGF-Trap in the presence of serum for 3 days. Shown in (**E**,**F**) are mean % (±SE) (top) and fold change (bottom) relative to control, vehicle-treated cells that were assigned a value of 100% and 1, respectively (*n* = 3). * *p* < 0.05, ** *p* < 0.01, *** *p* < 0.001.

Having established IGF-1 responsiveness in the MDA-MB-231 cells, we then analyzed the effect of the IGF-Trap on IGF signaling and functions. We observed that in the presence of the IGF-Trap, ligand mediated IGF-IR activation and downstream signaling were inhibited (Figure 2A–D). The IGF-Trap also inhibited MDA-MB-231 proliferation as assessed by the MTT assay (Figure 2E,F), establishing the sensitivity of these cells to IGF signaling blockade even in the presence of serum.

### 3.3. Increased Constitutive ERK Activation Contributes to an Acquired Resistance of MDA-MB-231 Cells to the IGF-Trap

**Figure 3:**
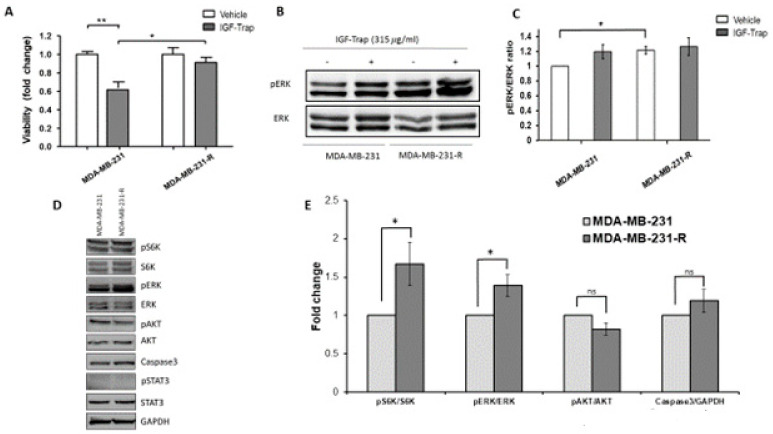
Acquired resistance to the growth inhibitory effect of IGF-Trap and augmented ERK signaling following sustained exposure of MDA-MB-231 to IGF-Trap in vitro. MDA-MB-231 exposed to gradually increasing concentrations of IGF-Trap for 3 months (MDA-MB-231-R) and the parental cells were treated with the IGF-Trap (315 μg/mL) for 72 h. Cell proliferation was measured using the MTT assay. Shown in (**A**) are means ± SE of the results (*n* = 5) expressed as fold change relative to the respective vehicle-treated controls that were assigned a value of 1. ERK activation (**B**) was analyzed by Western blotting. Shown in (**B**) is a representative immunoblot and in (**C**) the means ± SE (*n* = 3) expressed as fold change in the pERK/ERK ratio relative to vehicle-treated cells that were assigned a value of 1. Shown in (**D**) is a representative Western blot of additional signal transduction mediators analyzed in these cells, and in the bar graph (**E**) the means (±SE) of 3 experiments expressed as phosphorylated to total protein ratios normalized to the levels of parental MDA-MB-231 cells that were assigned a value of 1. * *p* < 0.05, ** *p* < 0.01, ns—not significant.

To further investigate potential mechanisms that could drive the emergence of resistant cells, as a consequence of long-term treatment with the IGF-Trap, we exposed MDA-MB-231 cells in vitro, over a period of 3 months, to gradually increasing concentrations of the IGF-Trap in serum-containing medium. This resulted in the emergence of a MDA-MB-231 subpopulation that was no longer sensitive to the IGF-Trap (MDA-MB-231-R) (Figure 3A), as measured using the MTT assay. Western blotting revealed in these cells increased basal levels of phospho-ERK that were no longer affected by the presence of IGF-Trap in the medium, indicative of increased constitutive activation of the MEK/ERK signaling pathway in these cells (Figure 3B,C). These results indicated that the loss of IGF-IR dependency in MDA-MB-231 cells subjected to long-term IGF-Trap treatment was associated with increased ERK activation, likely providing IGF-independent growth signals. We analyzed other potential changes in signaling pathways in these cells and found that S6K activation was also enhanced. However, no significant change was found in constitutive Akt activation levels, no activation of STAT3 was detectable and no change was observed in total caspase 3 levels (Figure 3D,E). Interestingly, while insensitive to IGF-Trap, exogenous IGF-1 could still activate the receptor in these cells, although the kinetics of the response were distinct from those seen with parental MDA-MB-231 cells (Supplementary Figure S1) and no additional ERK activation was observed.

### 3.4. Expression and Activation Levels of FGFR1 Are Upregulated in MDA-MB-231-R Cells

**Figure 4:**
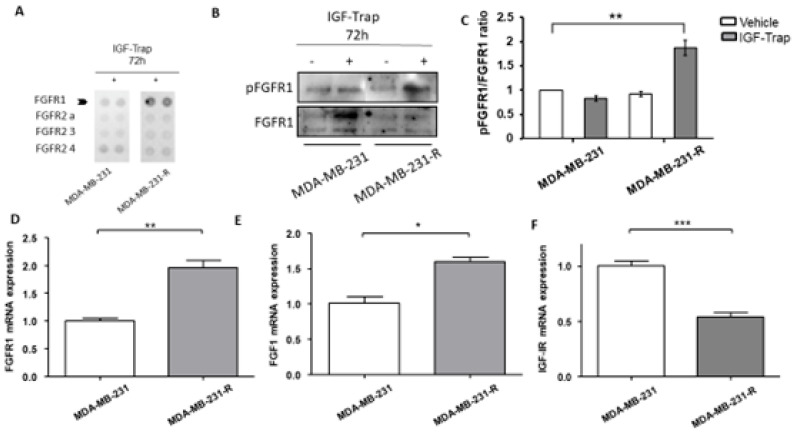
Upregulated expression and increased activation of FGFR1 in IGF-Trap resistant MDA-MB-231-R cells. MDA-MB-231 and MDA-MB-231-R cells were treated with 315 μg/mL IGF-Trap in the presence of serum for 72 h. A phopsho-RTK (receptor tyrosine kinase) array was then used (**A**) to profile phosphorylated RTKs in total cell lysates derived from these cells. Western blotting (**B**) and qPCR (**D**–**F**) were used to analyze activated FGFR1 (**B**) and mRNA expression (**D**–**F**) levels of the indicated transcripts in cells treated with 160 μg/mL IGF-Trap for 72 hr prior to lysis. Shown in (**B**) is a representative immunoblot and in (**C**) the data expressed as pFGFR1/FGFR1 ratios normalized to the level in vehicle-treated MDA-MB-231 cells that were assigned a value of 1. Data in (**D**–**F**) were normalized to GAPDH and are expressed as means (±SE) fold change in transcript expression in MDA-MB-231-R cells relative to MDA-MB-231 cells that were assigned a value of 1 (*n* = 4). * *p* < 0.05, ** *p* < 0.01, *** *p* < 0.001.

The increase in ERK activation in MDA-MB-231-R cells suggested that the resistance to the IGF-Trap was associated with increased activation of an alternate ERK-activating growth factor receptor. We therefore subjected the resistant and parental cells to analysis by a phospho-receptor tyrosine kinases (p-RTKs) array to identify potential changes in RTK activity in these cells. Of the 49 different human RTKs arrayed, FGFR1 was found to be preferentially activated in MDA-MB-231-R cells when compared to the parental line (Figure 4A) and this was confirmed by Western blotting, revealing a ~2-fold increase in phospho-FGFR1 in the resistant, relative to the parental cells (Figure 4B). Moreover, FGFR1 and its ligand FGF1 were significantly upregulated in MDA-MB-231-R cells (Figure 4C,D) as assessed by qPCR, while IGF-IR expression was downregulated (Figure 4E).

Of interest, the expression of the RTKs epidermal growth factor receptor (EGFR) and Met were also detected in the MDA-MB-231 cells by RT-PCR; however, the expression of the respective ligands was either non-detectable (hepatocyte growth factor—HGF) or very low (epidermal growth factor—EGF) (Supplementary Figure S2). Collectively, these data suggested that the acquired resistance to the IGF-Trap in the MDA-MB-231-R cells was associated with increased FGFR1 expression and activation, likely through autocrine FGFR1 signaling.

### 3.5. The Selective FGFR1 Inhibitor PD166866 Decreases Proliferation and Triggers Cell Cycle Arrest in MDA-MB-231 Cells

**Figure 5:**
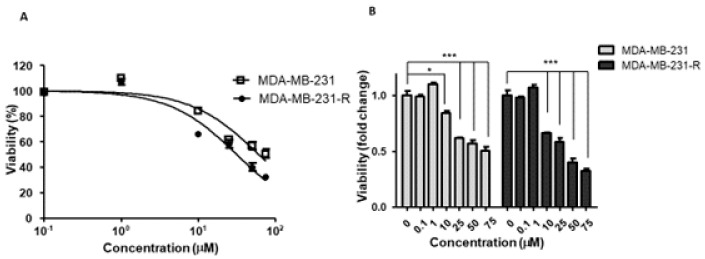
Increased sensitivity of MDA-MB-231-R cells to FGFR1 signaling blockade. Cells in complete medium were treated for 72 h with the FGFR1 inhibitor PD166866 and cell proliferation measured by the MTT assay. Shown in (**A**) is a dose response curve and in (**B**) a bar graph depicting the anti-proliferative effect of PD166866. Results are expressed as means ± SE (*n* = 3) relative to the respective, vehicle-treated controls that were assigned a value of 100% and 1, respectively. * *p* < 0.05, *** *p* < 0.001.

Having identified FGFR1 as the RTK that could potentially rescue MDA-MB-231 cells from the growth-inhibitory effect of the IGF-Trap, we sought to evaluate the sensitivity of these cells to FGFR1 inhibition and therefore treated both parental and resistant MDA-MB-231 cells with the FGFR1-selective, small molecule TKI PD166866, using a range of concentrations known to inhibit FGFR1 specifically [32]. We found that this inhibitor reduced the proliferation of both cell types in a dose-dependent manner (Figure 5A,B). However, MDA-MB-231-R cells exhibited a greater sensitivity to this inhibitor, as evidenced by a 2-fold lower IC_50_ as compared to the parental MDA-MB-231 cells (Figure 5A) and a greater decrease in proliferation at PD166866 concentrations of 10–75 μM (Figure 5B).

### 3.6. The Selective FGFR1 Inhibitor PD166866 Causes Cell Cycle Arrest in MDA-MB-231 Cells

**Figure 6:**
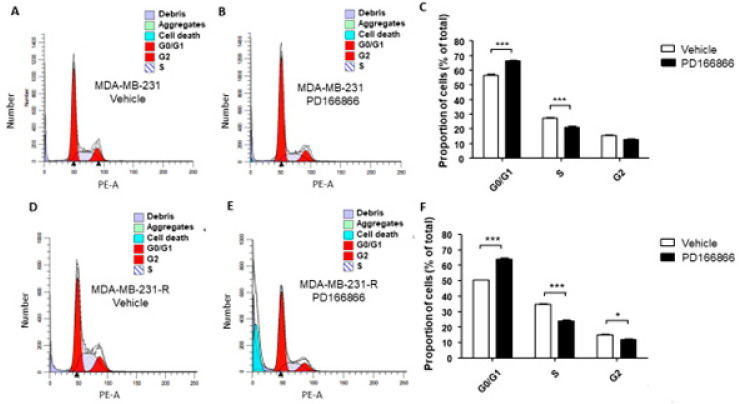

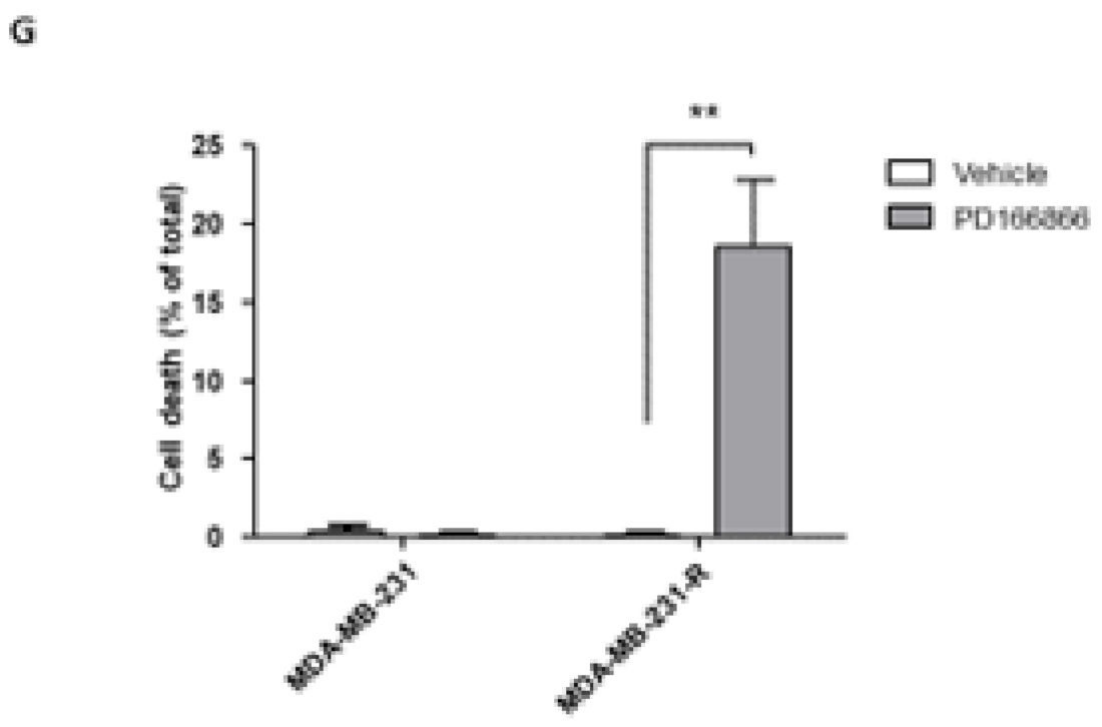
Cell cycle arrest in MDA-MB-231 and MDA-MB-231-R cells treated with PD166866. Cells were treated with 10 μM PD166866 or vehicle in complete medium for 72 h, fixed, stained with propidium iodide and cell cycle analysis performed by flow cytometry. Shown are representative flow cytometry histograms of MDA-MB-231 (**A**,**B**) and MDA-MB-231-R (**D**,**E**) cells. Shown in the bar graphs are the calculated proportions of cells at different cell cycle phases for MDA-MB-231 (**C**) and MDA-MB-231-R cells (**F**) and in (**G**) the proportions of dead cells for each cell type. Results are based on three independent experiments and are expressed as mean percentage (±SE) of total cells in (**C**,**F**) and percent of all events analyzed in (**G**). * *p* < 0.05, ** *p* < 0.01, *** *p* < 0.001.

The above results suggested that a sustained IGF-signaling blockade increased the growth-dependency of the surviving cells on FGFR1 signaling. This was also evident when the effect of this inhibitor on cell cycle progression was analyzed using flow cytometry. PD166866 induced cell cycle arrest in both MDA-MB-231 (Figure 6A–C) and MDA-MB-231-R (Figure 6D–F) cells, significantly lowering G_1_-S phase transition in both cell types (Figure 6A–F). Importantly, however, increased cell death was observed only in MDA-MB-231-R cells (Figure 6G), suggesting an increased dependency of these cells on FGFR1 signaling for survival.

### 3.7. FGFR1 Silencing Increases the Sensitivity of MDA-MB-231 Cells to the IGF-Trap

**Figure 7:**
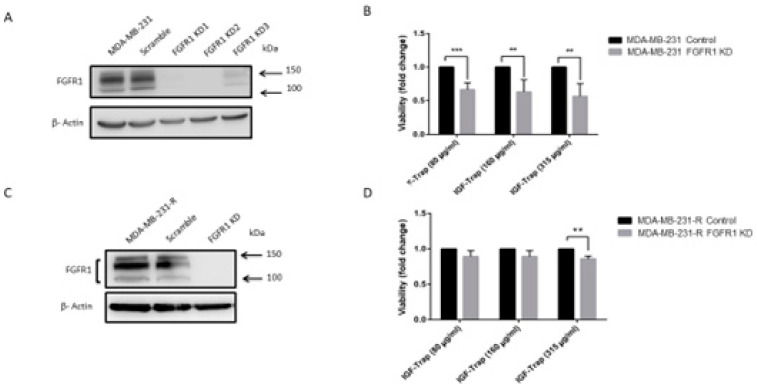
FGFR1 silencing enhances MDA-MB-231 cell sensitivity to the IGF-Trap. FGFR1 was silenced using lentiviral FGFR1 short hairpin RNA (shRNA). Shown in (**A**,**C**) are representative Western blots confirming FGFR1 silencing in MDA-MB-231 and MDA-MB-231-R cells, respectively. Cells were treated with the indicated concentrations of the IGF-Trap in the presence of serum for 48 hr and proliferation (**B**,**D**) measured using the colorimetric MTT assay. Data are expressed as means ± SE (*n* = 3), relative to controls that were assigned a value of 1. ** *p* < 0.01, *** *p* < 0.001.

Our data suggested that FGFR1 activation rendered MDA-MB-231 cells resistant to IGF-axis targeting, providing alternate growth and survival signaling. To further confirm that FGFR1 signaling was driving this acquired resistance, we silenced FGFR1 expression in MDA-MB-231 and MDA-MB-231-R cells using shRNA (Figure 7A,C) and analyzed the effect of FGFR1 loss on the sensitivity of these cells to IGF-Trap treatment. We first confirmed that FGFR1 silencing did not reduce cell proliferation in serum containing medium (Supplementary Figure S3). When cell proliferation was then measured in the presence of the IGF-Trap, we found a significant reduction (50–60%) in FGFR1-deficient wild type MDA-MB-231 cells, as compared to control cells that were transduced with a vector expressing a scrambled sequence (Figure 7B). The effect of FGFR1 silencing on MDA-MB-231-R proliferation, however, was more moderate, with a significant effect (15% reduction) seen only at the highest IGF-Trap concentration of 315 μg/mL (Figure 7D). Together, these results confirmed that FGFR1 signaling provided MDA-MB-231 cells with a growth advantage in the presence of IGF-axis blockade. In addition, the results also suggested that re-acquisition of sensitivity to the IGF-Trap in the resistant cells was likely multi-factorial. Intriguingly, we found that FGFR1 silencing increased IGF-1R, IGF-1 and IGF-2 expression levels in MDA-MB-231 but not in MDA-MB-231-R cells (Supplementary Figure S4), suggesting that increased total IGF-1R signaling may have contributed to the higher sensitivity of these cells to IGF-Trap.

### 3.8. Combinatorial Therapy Increases Cell Cycle Arrest in MDA-MB-231-R Cells

**Figure 8:**
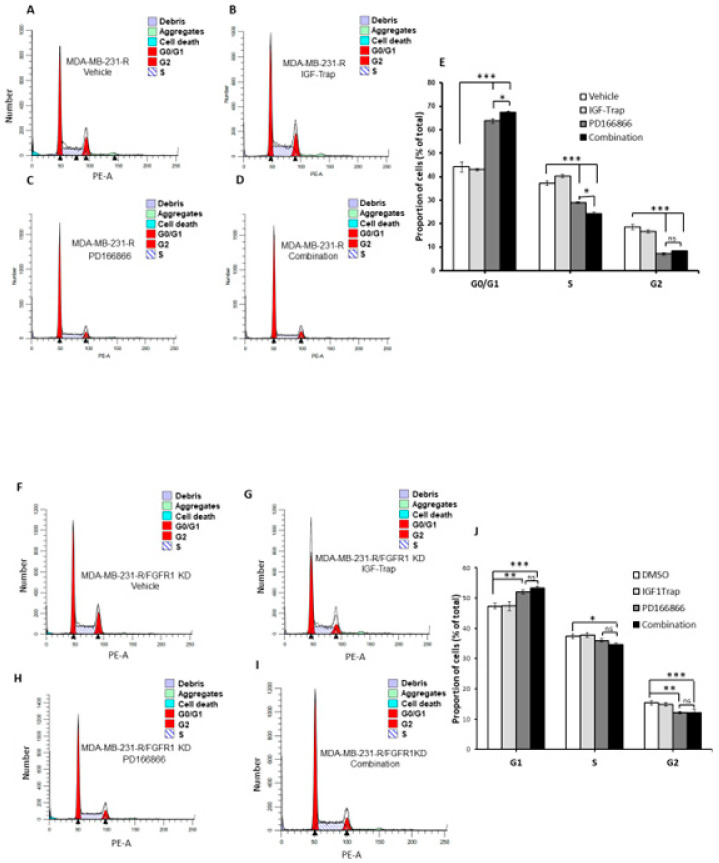
Combination FGFR and IGF-IR targeting enhances cell cycle arrest in MDA-MB-231-R cells. Cells were starved overnight and then cultured in DMEM medium containing 10% fetal bovine serum (FBS) with (or without) IGF-Trap (160 μg/mL), PD166866 (20 μM) or both for 48 h prior to cell cycle analysis. A single representative cell cycle profile is shown for MDA-MB-231-R (**A**–**D**) and MDA-MB-231-R cells in which FGFR1 was silenced using shRNA (MDA-MB-231-R/FGFR1 KD cells (**F*–*I**) treated with vehicle (**A**,**F**), IGF-Trap (**B**,**G**), PD166866 (**C**,**H**) or combination of the two (**D**,**I**). The proportions of cells (%) in the different cell cycle phases are shown in the bar graphs (**E**,**J**). * *p* < 0.05, ** *p* < 0.01, *** *p* < 0.001.

We also analyzed the effect of combinatorial IGF-1R and FGFR1 inhibition on cell cycle progression in the resistant cells and used MDA-MB-231-R cells in which FGFR1 was silenced as a specificity control. Cells were treated for 48 h with the indicated inhibitors (Figure 8) and cell cycle analysis was performed using flow cytometry. IGF-Trap treatment alone did not significantly affect cell cycle progression in either of these cells (Figure 8B,G), while PD166866 caused a much greater increase in cell cycle arrest at the G0/G1 phase in MDA-MB-231-R than in the FGFR1 knockdown cells (Figure 8C,E,H,J). This inhibitory effect on MDA-MB-231-R cells (but not MDA-MB-231 knockdown (KD) cells was further enhanced when the cells were treated with a combination of PD166866 and IGF-Trap (Figure 8D,E). Unexpectedly, PD166866 with or without IGF-Trap still had a minor effect on S/G2 transition in cells whose FGFR1 expression was silenced (Figure 8H–J).

### 3.9. FGFR1 Silencing Increases the Sensitivity of MDA-MB-231 Cells to IGF-Trap Treatment In Vivo

**Figure 9:**
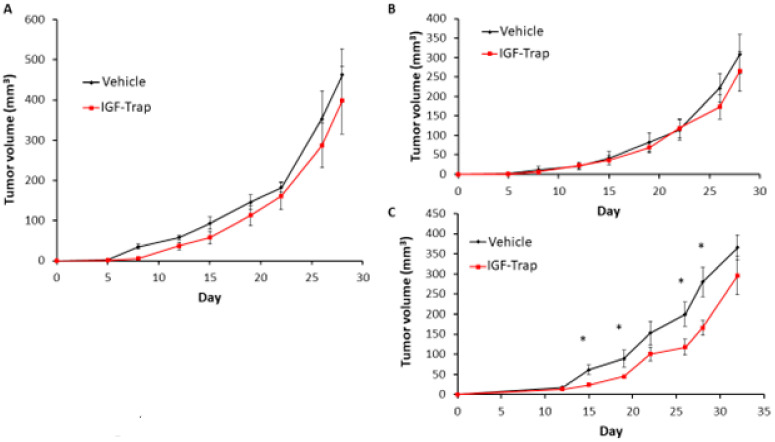
FGFR1 silencing increases the sensitivity of MDA-MB-231-R cells to IGF-Trap treatment in vivo. FGFR1-silenced MDA-MB-231-R cells in Matrigel were injected into the mammary fatpads of female NSG mice and the mice treated, twice weekly, with 5mg/kg IGF-Trap, intravenously. Tumor volumes were recorded twice weekly. Shown are mean tumor volumes (±SE) in mice injected with wild type (**A**), control transfected (**B**) and FGFR1 silenced (**C**) MDA-MB-231-R cells (*n* = 5). Note the initial delay in tumor appearance in mice injected with FGFR1 silenced MDA-MB-231 cells. * *p* < 0.05 as analyzed by the Student’s *t*-test.

Finally, to assess whether the silencing of FGFR1 affected the response of MDA-MB-231 cells to the IGF-Trap in vivo, we injected the FGFR1-silenced MDA-MB-231-R cells into the mammary fatpads of female NSG mice and injected the mice, twice weekly, with 5 mg/kg IGF-Trap, intravenously. Tumor growth in these mice was compared to growth in mice injected with untreated or control-transfected cells that were treated in the same manner. We observed a delay in tumor appearance in all mice injected with FGFR1-deficient cells and these cells grew at a slower rate than FGFR1-competent cells regardless of treatment (Figure 9A–C), suggesting the FGFR1 signaling contributed to tumor cell growth in the mammary fatpad. As expected, IGF-Trap treatment had no significant effect on the growth of either of the control MDA-MB-231-R cells (Figure 9A,B). However, in mice injected with FGFR1-silenced MDA-MB-231-R cells, there was a significant reduction in tumor growth rate as compared to mice injected with the vehicle (PBS) only (Figure 9C), for up to 30 days post tumor implantation. These results confirmed that FGFR1 enhanced the resistance of these cells to IGF-IR targeting in vivo.

## 4. Discussion

Disappointing results from clinical trials with agents that target the IGF axis [33,34,35] highlighted the need for predictive markers for patient selection. Consequently, there has been considerable effort to identify biomarkers of sensitivity to drugs that target the IGF-axis. As anti-cancer therapies that target a single RTK often result in the rapid emergence of resistant cells, the identification of molecular mechanisms that could provide compensatory growth signaling in the absence of IGF-IR activation is critical for the design of effective combination therapies.

**Figure 10:**
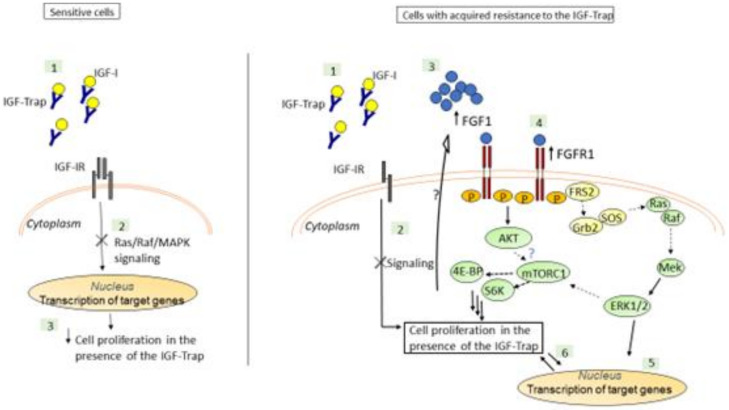
A postulated model for the mechanism underlying IGF-Trap resistance in MDA-MB-231. The IGF-Trap inhibits IGF-IR signaling, decreasing ERK phosphorylation and cell cycle progression and inducing apoptosis in MDA-MB-231 cells (**Left**). Our findings suggest that MDA-MB-231 cells that are continuously exposed to the IGF-Trap lose the sensitivity to the growth inhibitory effect of the Trap, due to increased autocrine FGFR1 signaling and ERK activation, mediated by increased FGFR1/FGF1 expression (**Right**). The increase in constitutive ERK phosphorylation may also contribute to increased mTORC1/S6K activation and protein synthesis in these cells, either directly or through crosstalk with the PI3K-AKT pathway [36]. Please note that postulated interactions are indicated by dotted arrows, while interactions elucidated by our data are indicated by solid arrows.

The IGF-Trap is a novel anti-cancer drug candidate [27]. We observed that human TNBC MDA-MB-231 xenografts had a range of susceptibility levels to the IGF-Trap in vivo [23], and our aim was to identify potential resistance mechanism(s) to the IGF-Trap following sustained exposure of these cells to the IGF-Trap in vitro. Consistent with other reports [18], we found that IGF-1 activated ERK and PI3K/Akt signaling in these cells and demonstrated that the IGF-Trap inhibited signaling and suppressed their proliferation in vitro in a dose-dependent manner. However, sustained exposure to the IGF-Trap resulted in the emergence of a subpopulation that was no longer sensitive to IGF-targeting, and this was associated with increased FGFR1 expression and signaling in these cells (see diagrammatic representation in Figure 10).

We found that the IGF-Trap-resistant cells were sensitive to a selective FGFR1 inhibitor that caused cell cycle arrest and cell death. Interestingly, when FGFR1 expression was silenced using shRNA, a marked increase in the anti-proliferative effect of IGF-Trap in vitro was observed in the parental MDA-MB-231 cells, but the resistant cells did not re-acquire the same sensitivity to IGF-Trap when the latter was used as a single agent. This may have been due to reduced IGF-IR expression levels in the MDA-MB-231-R cells (Figure 4) rendering their growth IGF-signaling independent. It is also conceivable that in addition to increased autocrine FGFR1 activation, these cells underwent other phenotypic changes that contributed to resistance to IGF-signaling inhibition.

We found that blockade of FGFR1 signaling using the chemical inhibitor PD166866 could induce cell cycle arrest and reduce cell proliferation in both MDA-MB-231 and MDA-MB-231-R cells, suggesting that sustained exposure to IGF-Trap likely resulted in the selection of pre-existing MDA-MB-231 clone(s) that were intrinsically FGF dependent for proliferation. Indeed, we found that clonal subpopulations of MDA-MB-231 expressed divergent levels of IGF-IR and FGFR1 (Supplementary Figures S5 and S6), and this may have determined their response to the IGF-Trap and the FGFR1 inhibitor. PD166866 induced cell death selectively in MDA-MB-231-R cells. This suggests that reduced IGF-1R expression and the loss of IGF-responsiveness in these cells increased their dependency on FGF signaling for survival and rendered them particularly sensitive to FGF signaling blockade. Consistent with our findings, Chen et al. have recently shown that PD166866 inhibited proliferation and triggered anoikis in FGFR1-amplified breast cancer cells and identified the suppression of the Akt/mTOR signaling pathway as the underlying mechanism [37]. Of interest, we observed increased S6K activation levels in MDA-MB-231-R cells, suggesting that increased autocrine FGFR1 signaling in these cells also enhanced mTORC1-S6K activation levels. This may have occurred downstream of constitutive ERK activation, via the AKT pathway or through crosstalk between these two signaling pathways (extensively reviewed in Mendoza et al. [36]). Interestingly, we also found that while ERK signaling was not altered in response to IGF-1 in MDA-MB-231-R cells, Akt phosphorylation was increased. This suggests that in these cells, autocrine FGFR1 signaling resulted in preferential activation of the ERK pathway, leading to loss of responsiveness of this pathway to IGF signaling. This may be due, at least partially, to the manner in which these IGF-Trap resistant cells were selected, namely a step-wise enrichment of clonal subpopulations that retained (ERK-dependent) proliferative capacity in the presence of the IGF-Trap. The role of the PI3K/Akt/mTOR pathway in our cells remains to be fully elucidated. It should also be noted that although, to our knowledge, IGF-IR has not been identified as a regulator of FGFR expression levels per se, the possibility that in our cells, the persistent IGF-1R signaling blockade played a direct or indirect role in regulating FGFR1 expression and activation levels [38] cannot, at present, be entirely ruled out. Indeed, crosstalk between the IGF and FGF axes has been documented in different cell types. For example, Shi et al. reported that FGFR1 overexpression induced the activation of IRS1 and IGF-1R in breast carcinoma cells [38], and in another study, ligand-activated FGFR increased the expression of IGFs in multipotent adult stem cells [39]. Intriguingly, in our model, we found an inverse correlation between FGFR1 expression/activation levels and the expression levels of IGF-1R and its ligands (Figure 4 and Supplementary Figure S4). This suggests that the crosstalk between these signaling axes may be context specific. In the present model, it may also reflect a clonal selection process, whereby reduced FGFR1 signaling preferentially selects clones with compensatory IGF-IR signaling, and vice versa.

We found that FGFR1 silencing significantly increased the sensitivity of MDA-MB-231-R cells to IGF-Trap treatment in vivo, although it could not completely inhibit tumor growth, over time. Namely, we observed that the growth rate of IGF-Trap treated FGFR1-deficient tumors was initially significantly lower than that of non-treated tumors, but eventually accelerated over time, despite continuous treatment. This may have been due to the proliferation and eventual dominance of subclones expressing low residual FGFR1 levels in this population. The existence of cells that retained some sensitivity to FGFR1 inhibition in this FGFR1 silenced population is also suggested by our cell cycle analysis (Figure 8J). Alternatively, clonal subpopulations with alternate resistance mechanisms may have emerged during the prolonged treatment. The complete elimination of potentially resistant clonal subpopulations may therefore require further optimization of the treatment or a combinatorial therapy with other drugs. In this regard, combining standard of care chemotherapy with IGF-Trap administration may be one potential strategy for achieving a more complete response.

FGFR was identified as a potential oncogenic driver in TNBC. Sharpe et al. [40] recently reported that autocrine FGFR signaling could promote the growth of TNBC cells in vitro and in vivo and *FGFR1/2* amplifications were identified in residual TNBC disease following neoadjuvant chemotherapy [41]. Interestingly, IGF-IR amplification was also observed in some of these samples.

While our study focused on a single TNBC cell line and the broader relevance of the resistance mechanism, we identified, remains to be confirmed in other TNBC models, our results are in line with similar findings in TNBC and other malignancies. For example, FGFR1 expression was identified as a predictor of poor overall survival in TNBC patients [42], and FGFR1 amplifications and overexpression have also been linked to de novo tamoxifen-resistance in luminal type breast cancer cell lines [43]. In the latter cells, increased ligand-dependent and ligand-independent FGFR1 activation and signaling were observed, and re-sensitization to tamoxifen was achieved when FGFR1 expression was silenced, suggesting that resistance to hormonal therapy could also be mediated by overexpression and/or activation of the FGF axis, and that dual targeting could provide protection from the emergence of resistant cells. FGFR overexpression was also shown to render breast carcinoma cells resistant to metformin, and this was also linked to ERK activation [38]. Of note, FGFR overexpression and activation were identified as a mechanism of resistance to IGF-1R inhibitors and other targeted drugs in other tumor types, including in a rhabdomyosarcoma model [44] and in lung cancer [45].

Taken together, the results suggest that combinatorial therapy with IGF-1R and FGFR inhibitors alone, or in combination with chemotherapy, could limit the emergence of resistant subpopulations in TNBC cells and optimize treatment efficacy.

## 5. Conclusions

Our results identify autocrine FGFR1 activation as a mechanism of MDA-MB-231 resistance to the anti-proliferative effects of a novel IGF-IR inhibitor. They suggest that co-targeting of the FGFR axis may potentiate the anti-cancer effect of IGF-1R inhibiting drugs.

## 6. Patents

The following patents were granted for the IGF-Trap technology:

(1) SOLUBLE IGF RECEPTOR Fc FUSION PROTEINS AND USES THEREOF Application No. 12857915.8 Case Ref. 05001770-467EP European Patent Office. Granted: February 2019.
(2) SOLUBLE IGF RECEPTOR Fc FUSION PROTEINS AND USES THEREOF—US—International (PCT) Patent Application No. PCT/CA2012/050899. United States. 2014-06-16. Granted: September 2019.

## Data Availability

The authors declare that the data supporting the findings of this study are available within the paper and its supplementary information files. Unprocessed (raw) data can be made available by the corresponding author upon reasonable request.

## Supplementary Materials

The following are available online at https://www.mdpi.com/article/10.3390/biom11040527/s1, Figure S1. Altered IGF-1R signaling in MDA-MB-231-R cells; Figure S2. Growth factors and receptors expressed in MDA-MB-231 cells; Figure S3. FGFR1 silencing does not alter MDA-MB-231 cell proliferation in complete medium; Figure S4. Increased IGF-1R and ligand expression in FGFR1 silenced MDA-MB-231 cell; Figure S5. Clonal populations of MDA-MB-231 cells have divergent IGF-1R expression levels; Figure S6. MDA-MB-231 clonal subpopulations have diverse FGFR1 expression levels.; Table S1: List of PCR primers used in this study; Table S2. Summary of growth factors and RTK expressed in MDA-MB-231 cells as determined by PCR analysis.

## References

[1] Ferlay J., Soerjomataram I., Dikshit R., Eser S., Mathers C., Rebelo M., Parkin D.M., Forman D., Bray F. (2014). Cancer incidence and mortality worldwide: Sources, methods and major patterns in GLOBOCAN 2012. Int. J. Cancer.

[2] Badve S., Dabbs D.J., Schnitt S.J., Baehner F.L., Decker T., Eusebi V., Fox S.B., Ichihara S., Jacquemier J., Lakhani S.R. (2011). Basal-like and tri-ple-negative breast cancers: A critical review with an emphasis on the implications for pathologists and oncologists. Mod. Pathol..

[3] Bianchini G., Balko J.M., Mayer I.A., Sanders M.E., Gianni L. (2016). Triple-negative breast cancer: Challenges and opportunities of a heterogeneous disease. Nat. Rev. Clin. Oncol..

[4] A Carey L., Winer E.P., Viale G., Cameron D., Gianni L. (2010). Triple-negative breast cancer: Disease entity or title of convenience?. Nat. Rev. Clin. Oncol..

[5] Kalimutho M., Parsons K., Mittal D., López J.A., Srihari S., Khanna K.K. (2015). Targeted Therapies for Triple-Negative Breast Cancer: Combating a Stubborn Disease. Trends Pharmacol. Sci..

[6] Bauer K.R., Brown M., Cress R.D., Parise C.A., Caggiano V. (2007). Descriptive analysis of estrogen receptor (ER)-negative, proges-terone receptor (PR)-negative, and HER2-negative invasive breast cancer, the so-called triple-negative phenotype: A popula-tion-based study from the California cancer Registry. Cancer.

[7] Lehmann B.D., A Bauer J., Schafer J.M., Pendleton C.S., Tang L., Johnson K.C., Chen X., Balko J.M., Gómez H., Arteaga C.L. (2014). PIK3CA mutations in androgen receptor-positive triple negative breast cancer confer sensitivity to the combination of PI3K and androgen receptor inhibitors. Breast Cancer Res..

[8] Morris G.J., Naidu S., Topham A.K., Guiles F., Xu Y., McCue P., Schwartz G.F., Park P.K., Rosenberg A.L., Brill K. (2007). Differences in breast carcinoma characteristics in newly diagnosed African-American and Caucasian patients: A single-institution compilation compared with the National Cancer Institute’s Surveillance, Epidemiology, and End Results database. Cancer.

[9] Stead L.A., Lash T.L., Sobieraj J.E., Chi D.D., Westrup J.L., Charlot M., Blanchard R.A., Lee J.C., King T.C., Rosenberg C.L. (2009). Triple-negative breast cancers are increased in black women regardless of age or body mass index. Breast Cancer Res..

[10] Lehmann B.D., Bauer J.A., Chen X., Sanders M.E., Chakravarthy A.B., Shyr Y., Pietenpol J.A. (2011). Identification of human tri-ple-negative breast cancer subtypes and preclinical models for selection of targeted therapies. J. Clin. Investig..

[11] Liedtke C., Mazouni C., Hess K.R., André F., Tordai A., Mejia J.A., Symmans W.F., Gonzalez-Angulo A.M., Hennessy B., Green M. (2008). Response to Neoadjuvant Therapy and Long-Term Survival in Patients with Triple-Negative Breast Cancer. J. Clin. Oncol..

[12] Brodt P., Samani A., Navab R. (2000). Inhibition of the type I insulin-like growth factor receptor expression and signaling: Novel strategies for antimetastatic therapy. Biochem. Pharmacol..

[13] Haisa M. (2013). The type 1 insulin-like growth factor receptor signalling system and targeted tyrosine kinase inhibition in cancer. J. Int. Med. Res..

[14] Samani A.A., Yakar S., LeRoith D., Brodt P. (2007). The role of the IGF system in cancer growth and metastasis: Overview and re-cent insights. Endocr. Rev..

[15] Seccareccia E., Brodt P. (2012). The role of the insulin-like growth factor-I receptor in malignancy: An update. Growth Horm. IGF Res..

[16] Pollak M.N., Schernhammer E.S., Hankinson S.E. (2004). Insulin-like growth factors and neoplasia. Nat. Rev. Cancer.

[17] Sachdev D., Yee D. (2007). Disrupting insulin-like growth factor signaling as a potential cancer therapy. Mol. Cancer Ther..

[18] Davison Z., de Blacquière G.E., Westley B.R., May F.E. (2011). Insulin-like Growth Factor-Dependent Proliferation and Survival of Triple-Negative Breast Cancer Cells: Implications for Therapy. Neoplasia.

[19] Singh S.K., Tan Q., Brito C., De León M. (2010). Insulin-like growth factors I and II receptors in the breast cancer survival disparity among African–American women. Growth Horm. IGF Res..

[20] Law J.H., Habibi G., Hu K., Masoudi H., Wang M.Y.C., Stratford A.L., Park E., Gee J.M.W., Finlay P., Jones H.E. (2008). Phosphorylated Insulin-Like Growth Factor-I/Insulin Receptor Is Present in All Breast Cancer Subtypes and Is Related to Poor Survival. Cancer Res..

[21] Bahhnassy A., Mohanad M., Shaarawy S., Ismail M.F., El-Bastawisy A., Ashmawy A.M., Zekri A.R. (2015). Transforming growth factor-beta, insulin-like growth factor I/insulin-like growth factor I receptor and vascular endothelial growth factor-A: Prog-nostic and predictive markers in triple-negative and non-triple-negative breast cancer. Mol. Med. Rep..

[22] Avnet S., Sciacca L., Salerno M., Gancitano G., Cassarino M.F., Longhi A., Zakikhani M., Carboni J.M., Gottardis M., Giunti A. (2009). Insulin Receptor Isoform A and Insulin-like Growth Factor II as Additional Treatment Targets in Human Osteosarcoma. Cancer Res..

[23] Buck E., Gokhale P.C., Koujak S., Brown E., Eyzaguirre A., Tao N., Rosenfeld-Franklin M., Lerner L., Chiu M.I., Wild R. (2010). Compensatory insulin receptor (IR) activation on inhibition of insulin-like growth fac-tor-1 receptor (IGF-1R): Rationale for cotargeting IGF-1R and IR in cancer. Mol. Cancer Ther..

[24] Buck E., Mulvihill M. (2011). Small molecule inhibitors of the IGF-1R/IR axis for the treatment of cancer. Expert Opin. Investig. Drugs.

[25] Vaniotis G., Moffett S., Sulea T., Wang N., Elahi S.M., Lessard E., Baardsnes J., Perrino S., Durocher Y., Frystyk J. (2018). Enhanced anti-metastatic bioactivity of an IGF-TRAP re-engineered to improve physicochemical properties. Sci. Rep..

[26] Wang N., Rayes R.F., Elahi S.M., Lu Y., Hancock M.A., Massie B., Rowe G.E., Aomari H., Hossain S., Durocher Y. (2015). The IGF-Trap: Novel Inhibitor of Carcinoma Growth and Metastasis. Mol. Cancer Ther..

[27] Chen Y.M., Qi S., Perrino S., Hashimoto M., Brodt P. (2020). Targeting the IGF-Axis for Cancer Therapy: Development and Valida-tion of an IGF-Trap as a Potential Drug. Cells.

[28] Honegger A., E Humbel R. (1986). Insulin-like growth factors I and II in fetal and adult bovine serum. Purification, primary structures, and immunological cross-reactivities. J. Biol. Chem..

[29] Burnier J.V., Wang N., Michel R.P., Hassanain M., Li S., Lu Y., Metrakos P., Antecka E., Burnier M.N., Ponton A. (2011). Type IV collagen-initiated signals provide survival and growth cues required for liver metastasis. Oncogene.

[30] Vaniotis G., Rayes R.F., Qi S., Milette S., Wang N., Perrino S., Bourdeau F., Nyström H., He Y., Lamarche-Vane N. (2018). Collagen IV-conveyed signals can regulate chemokine production and promote liver metastasis. Oncogene.

[31] Seccareccia E., Pinard M., Wang N., Li S., Burnier J., Dankort D., Brodt P. (2014). The inhibitor of kappa B kinase-epsilon regulates MMP-3 expression levels and can promote lung metastasis. Oncogenesis.

[32] Risuleo G., Ciacciarelli M., Castelli M., Galati G. (2009). The synthetic inhibitor of fibroblast growth factor receptor PD166866 con-trols negatively the growth of tumor cells in culture. J. Exp. Clin. Cancer Res..

[33] Fuchs C.S., Azevedo S., Okusaka T., Van Laethem J.-L., Lipton L.R., Riess H., Szczylik C., Moore M.J., Peeters M., Bodoky G. (2015). A phase 3 randomized, double-blind, placebo-controlled trial of ganitumab or placebo in combination with gemcitabine as first-line therapy for metastatic adenocarcinoma of the pancreas: The GAMMA trial. Ann. Oncol..

[34] Langer C.J., Novello S., Park K., Krzakowski M., Karp D.D., Mok T., Benner R.J., Scranton J.R., Olszanski A.J., Jassem J. (2014). Randomized, Phase III Trial of First-Line Figitumumab in Combination with Paclitaxel and Carboplatin Versus Paclitaxel and Carboplatin Alone in Patients With Advanced Non–Small-Cell Lung Cancer. J. Clin. Oncol..

[35] Scagliotti G.V., Bondarenko I., Blackhall F., Barlesi F., Hsia T.C., Jassem J., Milanowski J., Popat S., Sanchez-Torres J.M., Novello S. (2015). Randomized, phase III trial of figitumumab in combina-tion with erlotinib versus erlotinib alone in patients with nonadenocarcinoma nonsmall-cell lung cancer. Ann. Oncol..

[36] Mendoza M.C., Er E.E., Blenis J. (2011). The Ras-ERK and PI3K-mTOR pathways: Cross-talk and compensation. Trends Biochem. Sci..

[37] Chen Y., Xie X., Li X., Wang P., Jing Q., Yue J., Liu Y., Cheng Z., Li J., Song H. (2016). FGFR antagonist in-duces protective autophagy in FGFR1-amplified breast cancer cell. Biochem. Biophys. Res. Commun..

[38] Shi Y., Ma Z., Cheng Q., Wu Y., Parris A.B., Kong L., Yang X. (2021). FGFR1 overexpression renders breast cancer cells resistant to metformin through activation of IRS1/ERK signaling. Biochim. Biophys. Acta (BBA) Bioenergy.

[39] Park S.-B., Yu K.-R., Jung J.-W., Lee S.-R., Roh K.-H., Seo M.-S., Park J.-R., Kang S.-K., Lee Y.-S., Kang K.-S. (2009). bFGF enhances the IGFs-mediated pluripotent and differentiation potentials in multipotent stem cells. Growth Factors.

[40] Sharpe R., Pearson A., Herrera-Abreu M.T., Johnson D., Mackay A., Welti J.C., Natrajan R., Reynolds A.R., Reis-Filho J.S., Ashworth A. (2011). FGFR Signaling Promotes the Growth of Triple-Negative and Basal-Like Breast Cancer Cell Lines Both In Vitro and In Vivo. Clin. Cancer Res..

[41] Balko J.M., Giltnane J.M., Wang K., Schwarz L.J., Young C.D., Cook R.S., Owens P., Sanders M.E., Kuba M.G., Sánchez V. (2014). Molecular Profiling of the Residual Disease of Triple-Negative Breast Cancers after Neoadjuvant Chemotherapy Identifies Actionable Therapeutic Targets. Cancer Discov..

[42] Cheng C.L., Thike A.A., Tan S.Y.J., Chua P.J., Bay B.H., Tan P.H. (2015). Expression of FGFR1 is an independent prognostic factor in triple-negative breast cancer. Breast Cancer Res. Treat..

[43] Turner N., Pearson A., Sharpe R., Lambros M., Geyer F., Lopez-Garcia M.A., Natrajan R., Marchio C., Iorns E., Mackay A. (2010). FGFR1 Amplification Drives Endocrine Therapy Resistance and Is a Therapeutic Target in Breast Cancer. Cancer Res..

[44] Huang F., Hurlburt W., Greer A., Reeves K.A., Hillerman S., Chang H., Fargnoli J., Finckenstein F.G., Gottardis M.M., Carboni J.M. (2010). Differential Mechanisms of Acquired Resistance to Insulin-like Growth Factor-I Receptor Antibody Therapy or to a Small-Molecule Inhibitor, BMS-754807, in a Human Rhabdomyosarcoma Model. Cancer Res..

[45] Manchado E., Weissmueller S., Morris J.P., Chen C.-C., Wullenkord R., Lujambio A., De Stanchina E., Poirier J.T., Gainor J.F., Corcoran R.B. (2016). A combinatorial strategy for treating KRAS-mutant lung cancer. Nat. Cell Biol..

