# Supplementary material for: Identification of a Resistance Mechanism to IGF-IR Targeting in Human Triple Negative MDA-MB-231 Breast Cancer Cells
Source: Biomolecules. 2021 Apr 1;11(4):527. doi: 10.3390/biom11040527 (PMC8065809; doi:10.3390/biom11040527)
Supplement: Supplementary file 1 [file biomolecules-11-00527-s001.pdf]

biomolecules

Supplementary materials

Identification of a Resistance Mechanism to IGF-IR Targeting in human Triple Negative MDA-MB-231 Breast Cancer Cells

Jennifer Tsui [1,2,†], Shu Qi [3], Stephanie Perrino [3], Matthew Leibovitch [3] and Pnina Brodt [1,2,3,4,5,*]

1 Department of Medicine, McGill University, Montreal, QC H4A 3J1, Canada; jennifer.tsui3@mail.mcgill.ca
2 Division of Experimental Medicine, McGill University, Montreal, QC H4A 3J1, Canada
3 McGill University Health Center Research Institute, Montreal, QC H4A 3J1, Canada; qishu2014@hotmail.com (S.Q.); stephanie.perrino@affiliate.mcgill.ca (S.P.); matthewleibovitch@hotmail.com (M.L.)
4 Department of Surgery, McGill University, Montreal, QC H4A 3J1, Canada
5 Department of Oncology, McGill University, Montreal, QC H4A 3J1, Canada
* Correspondence: pnina.brodt@mcgill.ca
† Current address: School of Veterinary Medicine, Murdoch University, Murdoch, WA 6150, Australia.

Citation: Tsui, J.; Qi, S.; Perrino, S.; Leibovitch, M.; Brodt, P. Identification of a Resistance Mechanism to IGF-IR Targeting in Human Triple Negative MDA-MB-231 Breast Cancer Cells. *Biomolecules* **2021**, *11*, 527. https://doi.org/10.3390/biom11040527

Received: 17 December 2020
Accepted: 29 March 2021
Published: 1 April 2021

Publisher's Note: MDPI stays neutral with regard to jurisdictional claims in published maps and institutional affiliations.

Copyright: © 2021 by the authors. Submitted for possible open access publication under the terms and conditions of the Creative Commons Attribution (CC BY) license (http://creativecommons.org/licenses/by/4.0/).

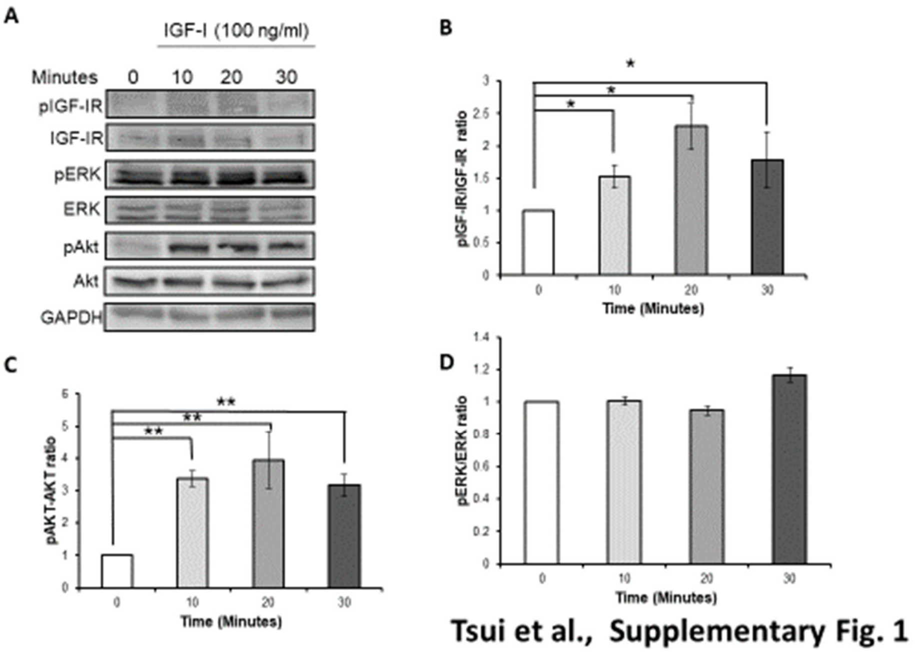

Figure S1. Altered IGF-1R signaling in MDA-MB-231-R cells. MDA-MB-231-R cells were serum starved for 24 hours then stimulated with 100 ng/ml IGF-1 for the indicated time intervals. Shown in **(A)** is a representative Western blot of the IGF-1R and downstream signaling mediators following IGF-1 stimulation (n=3). Shown in the bar graphs **(B-D)** are the mean (±SE) of 3 experiments expressed as phospho:total protein ratios normalized to the levels in unstimulated cells that were assigned a value of 1. *$p<0.05$, **$p<0.01$, ***$p<0.001$.

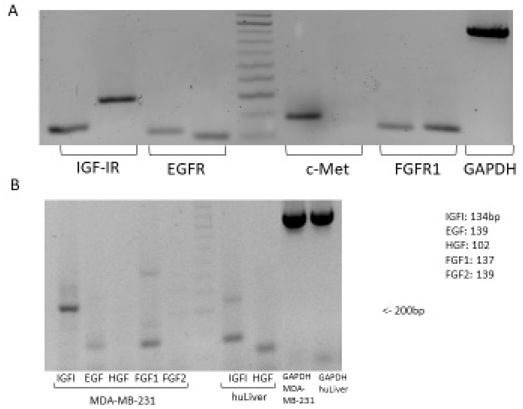

Tsui et al., Supplementary Fig 2

Figure S2. Growth Factors and receptors expressed in MDA-MB-231 cells. Shown are results of RT-PCR analyses for RTK (**A**) and growth factors (**B**) expressed in MDA-MB-231 cells. Two primer sets were used for each RTK (A). Human liver tissue (huLiver) was used as positive control in (B). The expected transcript size is indicated on the right.

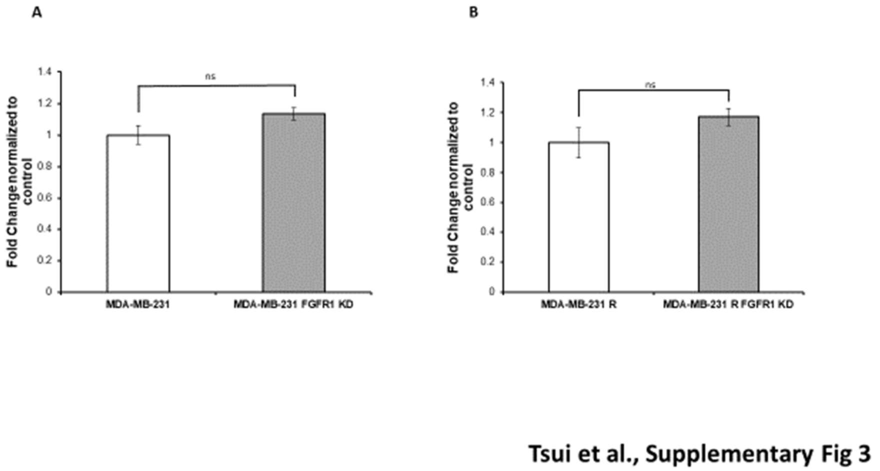

Figure 3. FGFR1 silencing does not alter MDA-MB-231 cell proliferation in complete medium. FGFR1 was silenced using lentiviral FGFR1 shRNA. Shown in (**A** and **B**) are results of a comparison of the increase in cell number following culture of the cells in complete medium for 48 hours as measured by the Trypan blue exclusion dye. The data are expressed as means ± SE (n=3), relative to the respective parental cells that were assigned a value of 1.

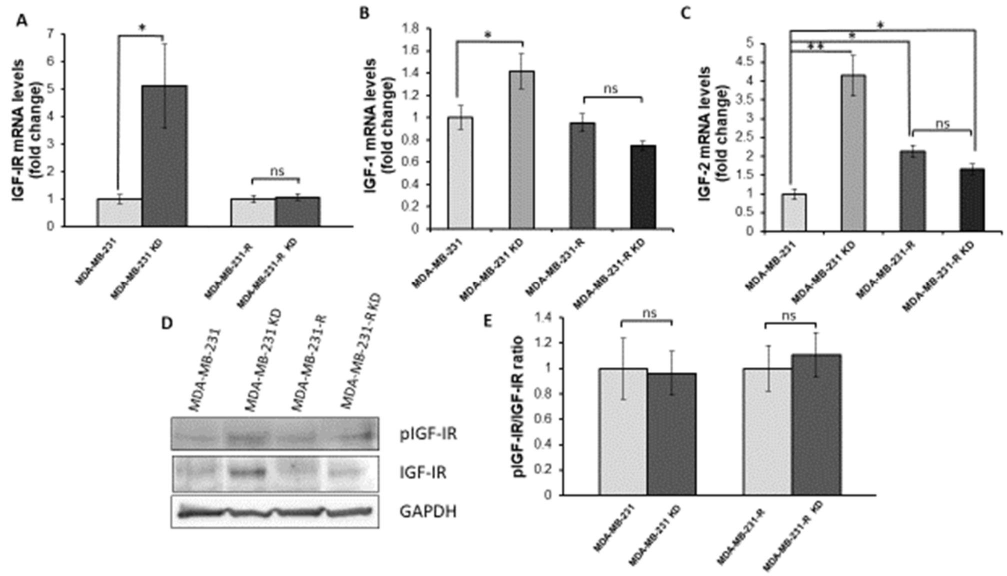

Figure S4. Increased IGF-1R and ligand expression in FGFR1 silenced MDA-MB-231 cells. Cells were cultured in complete media for 48 hours. Shown in **(A-C)** are results of qPCR for the indicated transcripts and in **(D)** results of Western blotting performed on the indicated cells. Shown in all bar graphs are means (± SE) of 3 experiments expressed relative to the levels measured in the respective parental cells that were assigned a value of 1. *$p<0.05$, **$p<0.01$, ***$p<0.001$.

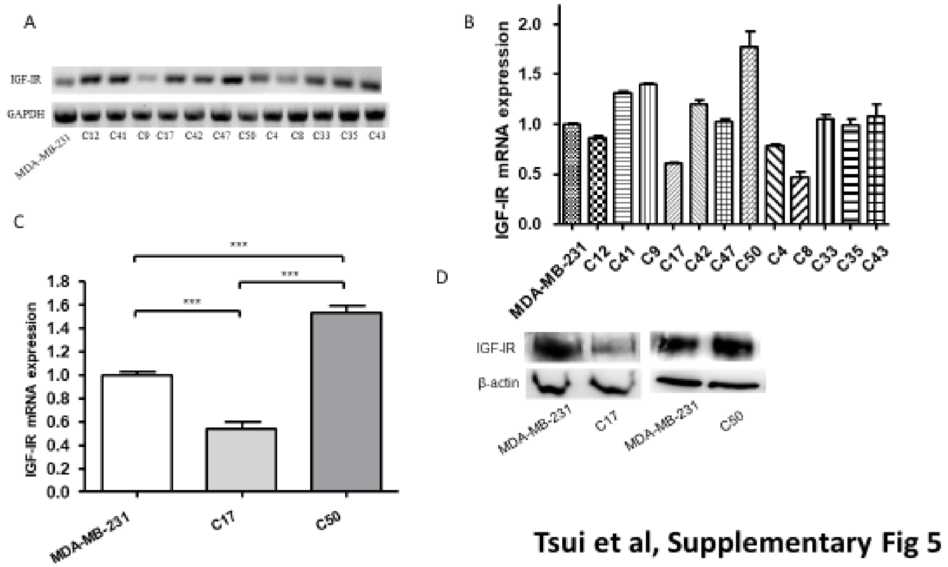

Figure 5. Clonal populations of MDA-MB-231 cells have divergent IGF-1R expression levels. MDA-MB-231 cells were clone by limiting dilution and IGF-1R expression levels in clonal subpopulations analyzed by RT-PCR. Shown in (**A**) are representative RT-PCR results of 2 independent experiments and in (**B**) expression levels normalized to GAPDH and expressed relative to parental MDA-MB-231 cells that were assigned a value of 1. qPCR was performed on mRNA extracted from low and high IGF-IR expressing clonal populations C17 and C50, respectively (**C**) and protein levels in these clones analyzed by Western blotting (**D**). Data in (**C**) were normalized to GAPDH and are expressed as means ± SE (n=4). ***$p < 0.001$.

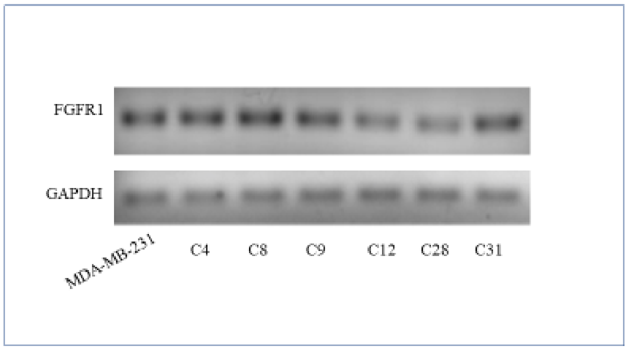

Tsui et al., Supplementary Fig 6

Figure S6. MDA-MB-231 clonal subpopulations have diverse FGFR1 expression levels. FGFR1 expression levels in clonal subpopulations of MDA-MB-231 (isolated as described in the legend to supplementary Figure 5) were measured by RT-PCR. Shown are representative results of 2 analyses.

Table S1. List of PCR primers used in this study. .

Gene	Primer Sequence
IGF-1R Forward (pair 1)	CCTGCACAACTCCATCTTCGTG
IGF-1R Reverse (pair 1)	CGGTGATGTTGTAGGTGTCTGC

IGF-1R Forward (pair 2)	ACGCCAATAAGTTCGTCCACAGAGACCT
IGF-1R Reverse (pair 2)	GAAGACTCCATCCTTGAGGGACTCAG
EGFR Forward (pair 1)	AACACCCTGGTCTGGAAGTACG
EGFR Reverse (pair 1)	TCGTTGGACAGCCTTCAAGACC
EGFR Forward (pair 2)	ACCTGCGTGAAGAAGTGTCC
EGFR Reverse (pair 2)	CGTCTTCCTCCATCTCATAGC
Met Forward (pair 1)	TGCACAGTTGGTCCTGCCATGA
Met Reverse (pair 1)	CAGCCATAGGACCGTATTTCGG
Met Forward (pair 2)	ATTTTGCTTTGCCAGTGGTGG
Met Reverse (pair 2)	GAGCGATGTTGACATGCCACT
FGFR1 Forward (pair 1)	GCACATCCAGTGGCTAAAGCAC
FGFR1 Reverse (pair 1)	AGCACCTCCATCTCTTTGTCGG
FGFR1 Forward (pair 2)	CACCCGAGGCATTATTTGAC
FGFR1 Reverse (pair 2)	AAGTTCCTCCACAGGCACAC
IGF-1 Forward	CTCTTCAGTTCGTGTGTGGAGAC
IGF-1 Reverse	CAGCCTCCTTAGATCACAGCTC
EGF Forward	TGCGATGCCAAGCAGTCTGTGA
EGF Reverse	GCATAGCCCAATCTGAGAACCAC
HGF Forward	GAGAGTTGGGTTCTTACTGCACG
HGF Reverse	CTCATCTCCTCTTCCGTGGACA
FGF1 Forward	ATGGCACAGTGGATGGGACAAG
FGF1 Reverse	TAAAAGCCCGTCGGTGTCCATG
FGF2 Forward	AGCGGCTGTACTGCAAAAACGG
FGF2 Reverse	CCTTTGATAGACACAACTCCTCTC
GAPDH Forward (PCR)	GGTGAAGGTCGGTGTGAACG
GAPDH Reverse (PCR)	AATGCCAAAGTTGTCATGGA
GAPDH Forward (qPCR)	TGCACCACCAACTGCTTAGC
GAPDH Reverse (qPCR)	GGCATGGACTGTGGTCATGAG

Table 2. Summary of growth factors and RTK expressed in MDA-MB-231 cells as determined by PCR analysis.

Receptors		Growth factors	
IGF-IR	+	IGF-I	-
EGFR	+	EGF	Low
Met	+	HGF	-
FGFR1	+	FGF1	+
		FGF2	-
